# Safe and effective use of nivolumab for treating lung adenocarcinoma associated with sporadic lymphangioleiomyomatosis: a rare case report

**DOI:** 10.1186/s12890-018-0775-5

**Published:** 2019-01-11

**Authors:** Johan Pluvy, Solenn Brosseau, Sandrine Stelianides, Claire Danel, Marina Nguenang, Antoine Khalil, Bruno Crestani, Gérard Zalcman, Valérie Gounant

**Affiliations:** 1 Service d’Oncologie Thoracique, Hôpital Bichat, 46 rue Henri Huchard, 75018 Paris, France; 2 CIC INSERM 1425-CLIP2 Paris-Nord, Hôpital Bichat-Claude Bernard, 46 rue Henri Huchard 75018, AP-HP, Paris, France; 3 Service de Réhabilitation Respiratoire, Hôpital Bichat, 46 rue Henri Huchard, 75018 Paris, France; 4 Service d’Anatomie Pathologique, Hôpital Bichat, 46 rue Henri Huchard, 75018 Paris, France; 5 Service de Radiologie, Hôpital Bichat, 46 rue Henri Huchard, 75018 Paris, France; 6 Service de Pneumologie, Hôpital Bichat, 46 rue Henri Huchard, 75018 Paris, France

**Keywords:** NSCLC, Sporadic lymphangioleiomyomatosis, Adenocarcinoma, Nivolumab, Immune checkpoint inhibitors

## Abstract

**Background:**

Sporadic lymphangioleiomyomatosis (LAM) is a rare form of diffuse parenchymal lung disease. PD-1 blocking antibodies constitute an essential treatment option for advanced non-small-cell lung cancer (NSCLC). The effect of immune checkpoint inhibitors in lymphangioleiomyomatosis patients with non-small cell lung cancer is unknown: concomitant symptomatic interstitial lung disease or the use of immunosuppressors was a key exclusion criterion in the original studies of immune checkpoint inhibitors, especially regarding the risk of interstitial lung disease exacerbation.

**Case presentation:**

A 48-year-old female, active smoker (36 pack-years), diagnosed with sporadic LAM since 2004 suffered from metastatic adenocarcinoma of the lung. Third-line therapy with nivolumab was started in 2015, with a major partial response. Due to pulmonary function alterations, sirolimus was also reinitiated in 2017 in conjunction with nivolumab, without any undesirable effects and a major partial response continuing up to May 2018.

**Conclusions:**

This case highlights the safe and effective use of nivolumab for managing metastatic lung adenocarcinoma that occurred in a patient with sporadic LAM. In the current case, immunotherapy proved highly successful in managing the NSCLC tumor that occurred upon LAM follow-up, with both a significantly prolonged partial response and acceptable safety profile.

## Background

Sporadic lymphangioleiomyomatosis (LAM) is a rare form of diffuse parenchymal lung disease occurring in women during reproductive years. Its clinical features include lung parenchymal cysts, chylous effusion, and recurrent pneumothorax, leading to progressive lung function loss due to lung destruction [1–4]. This condition may likewise be associated with extrapulmonary disease, such as abdominal lymphangioleiomyomas or renal angiomyolipomas with a risk of bleeding [1, 2].

Lung cancer is the leading cause of cancer death among women in developed countries [5]. Immune checkpoint inhibitors like PD-1 blocking antibodies constitute a new treatment option for advanced non-small-cell lung cancer (NSCLC) [6–8]. Concomitant symptomatic interstitial lung disease or the use of immunosuppressors was a key exclusion criterion in the original studies, owing to the risk of autoimmune lung disease with drugs that target the immune system [6–8].

LAM shares several features with cancer, such as estrogen receptor overexpression and dysregulation of the mammalian target of rapamycin (mTOR) pathway, leading to inappropriate proliferation, lymphangiogenesis, angiogenesis, and protease-driven matrix degradation [2]. Currently considered as a true tumor disease, this condition is defined as a subset of the perivascular epithelioid cell tumors, also known as the PEComas [9]. The mTOR inhibitors that target the mTOR pathway like sirolimus and everolimus have been proposed as potential treatment strategy and thus, an alternative to the anti-estrogen drugs commonly used in this disease [1].

The effect of immune checkpoint inhibitors in LAM patients is still unknown, especially regarding the risk of interstitial lung disease exacerbation. We report herein a case illustrating the safe and effective use of nivolumab for managing metastatic lung adenocarcinoma that occurred in a patient with sporadic LAM.

## Case presentation

A 48-year-old female, active smoker (36 pack-years) and without any occupational or environmental exposure, had been followed up for sporadic LAM since 2004. In her case, LAM was not associated with tuberous sclerosis complex. Initial computed tomography (CT) of the chest revealed diffuse bilateral cysts with thin walls that are typical of LAM, in addition to retroperitoneal involvement with left iliac, hypogastric, and latero-aortic angiomyolipomas. In April 2004, a biopsy of a retroperitoneal mass was performed revealing fusiform proliferation of smooth muscle-differentiated cells within a rich vascular and adipose stroma, with strong positivity for HMB45 staining, evocative of an angiomyolipoma. In 2006, the patient developed New York Heart Association Class II dyspnea on exercise, along with a chronic cough. From 2006 to 2007, she received several sequential anti-estrogen treatments, specifically tamoxifen and letrozole combined with triptorelin, with stable respiratory function. In 2007, the patient exhibited lung function deterioration, which led to the prescription of the mTOR inhibitor sirolimus (2 mg once daily, while the daily dose for treating renal cancer is 10 mg daily), resulting in the disappearance of retroperitoneal lesions.

**Fig. 1:**
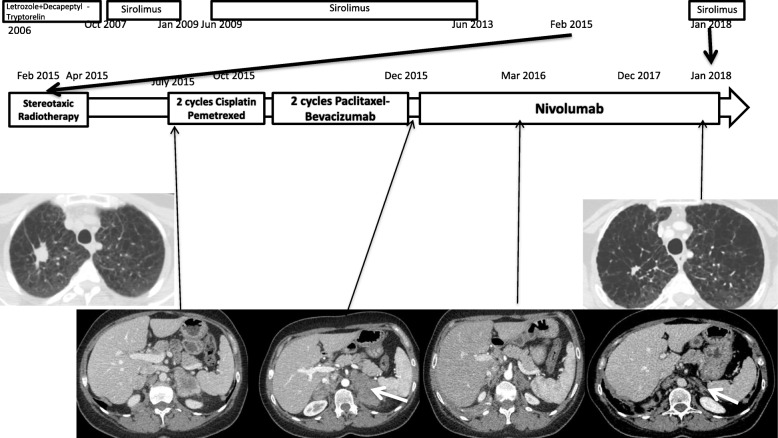
Timeline of cancer treatments from February 2015 to January 2018 with radiologic findings. Arrowheads showing left adrenal gland metastasis before initiation of Nivolumab (Dec 2015) and at 24 months with important partial response (Jan 2018)

In 2013, CT showed a right apical lung mass, highly suggestive of cancer, due to its size, radiological features, and hypermetabolism (SUVmax = 4.8) on TEP-CT. Sirolimus was stopped owing to its immunosuppressive effect, which may have induced cancer development. First, a CT-guided biopsy was then performed despite pulmonary functional impairment, with pathological analysis revealing neither tumoral lesion nor LAM cells, but rather fibroelastosic scarring. The decision to monitor CT without immediately repeating transthoracic biopsy was made, owing to the very small lesion size in a patient with functional impairment. For this reason, we thought that performing such a biopsy would have been too risky (Fig. 1).

Upon follow-up in 2015, due to the target lesions’ further growing while, another CT biopsy was carried out, showing mucin-producing adenocarcinoma (CK7+, CK20-, TTF1-) along with inflammatory stroma. EGFR and ALK testing proved negative. Additional molecular analyses revealed only a potentially oncogenic B-RAF mutation (c1406G > T; p.Gly469Val; COSM459) in exon 11. No c-met skip exon 14, PIK3CA, KRAS, or HER2 mutations were observed. PD-L1 immunohistochemistry testing was negative. No other distant lesion was observed. Cyberknife treatment of the lung lesion was carried out in April 2015.

However, a new patient assessment in July 2015 disclosed distant relapse in adrenal glands and left iliac lymph node, despite the thoracic lesion’s partial response. Due to high evidence of relapse on imaging, another tumor biopsy was not performed at that time. A first-line chemotherapy comprising cisplatin plus pemetrexed was initiated. As reassessment after two cycles in November 2015 showed local progression, a second-line treatment of weekly paclitaxel plus bevacizumab was initiated. In December 2015, performance status worsened from 1 to 2, with tumor assessment showing further progression in the adrenal glands.

Third-line therapy with nivolumab was started on December 11, 2015, given that no other viable treatment options were available. PD-L1 staining was negative at diagnosis and not tested again at relapse. Within 2 h of the first infusion, the patient developed complete right pneumothorax requiring pleural drainage. The persistence of pneumothorax at 48 h justified surgical pleurodesis, along with a 2-month hospitalization in the thoracic surgery department, owing to prolonged air leaking. A CT scan conducted 20 days after the only nivolumab infusion demonstrated a dramatic partial response in the lung and adrenal lesions (Fig. 1). Given that the pneumothorax occurred very shortly after nivolumab infusion, we hypothesized that the complication would more likely have been caused by LAM, rather than being directly related to nivolumab. Accordingly, we resumed nivolumab in January 2016, with no other recurrence of this adverse event.

Since that time the patient’s performance status has been 1 and she proved able to undergo pulmonary rehabilitation while gradually resuming work. Treatment was well tolerated with no major safety issues. The patient developed Grade II hypothyroidism treated with hormone replacement therapy. Lung function tests and symptoms worsened progressively from June 2013 to May 2017 (decline of CVF from 3700 mL in June 2013 to 2250 mL in May 2017, the decline being progressive). A bronchoalveolar lavage was performed showing no evidence of infection, tumoral cells, or lymphocytosis. This worsening was not accounted for by infection, tumor progression, or visible nivolumab-associated pneumonitis upon CT-scan follow-up. The patient’s deterioration was, therefore, attributed to LAM. While we observed a decline in lung function tests, there was no accelerated decline of lung function tests during nivolumab treatment, nor was there any argument for a LAM exacerbation.

In May 2017, sirolimus, stopped since 2013, was reinitiated in conjunction with nivolumab. It was a collective decision that was shared with the patient, because of the ethical challenge raised due to the lack of scientifically-sound evidence. CT-scans were performed every 2 to 3 months, and lung function tests every 6 months.”

**Fig. 2:**
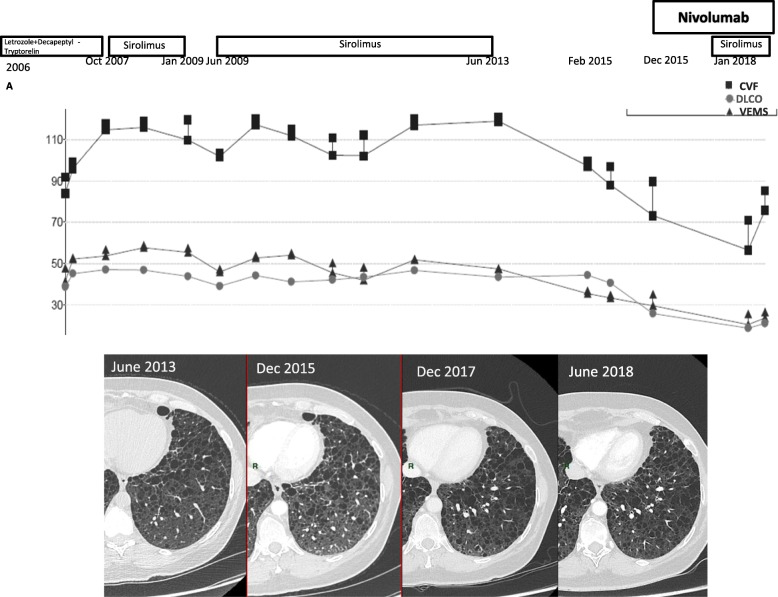
Evolution of pulmonary function tests from 2006 to January 2018 with corresponding timeline of treatments received for sporadic LAM. Radiologic findings in 2013, in 2015 before initiation of Nivolumab. In 2017 before addition of Sirolimus to Nivolumab and in June 2018 during association with nivolumab and sirolimus

She presented without any undesirable effects with improving lung function tests and a good partial response continuing up to February 2018 (Fig. 2).

## Discussion and conclusion

LAM is a rare cause of diffuse parenchymal lung disease [10]. Interstitial lung diseases have been a key exclusion criterion in original immune checkpoint inhibitor studies in NSCLC. While LAM displays the clinical and imaging pattern of interstitial lung disease, it is now deemed to display a neoplasm-like molecular pathogenesis. This neoplasm like pathogenesis was one of the reasons we thought that using immune checkpoint inhibition could be safe, despite the lack of published data in the literature and a possible risk of flare up of LAM. We had weighed up the risks and benefits before using immunotherapy for lung cancer treatment, prior to proposing this treatment strategy to the patient. Our decision proved to be congruent with the recent expert review by Postow et al. [11].

According to Klarquist [12] and Carbone [13], LAM cells bear antigenic similarities with melanoma cells, such as sharing several common immune markers. To illustrate, some LAM cells can express gp100 and other melanoma antigens (MART-1) that tend to be recognized by T-cells. Thus, the authors hypothesized that immunotherapy successfully developed against melanoma could constitute a reasonable treatment approach for LAM, despite interstitial lung disease commonly being the disease presentation mode.

According to Maisel et al. [14], PD-L1 is up-regulated in human lung tissue with LAM and in a murine model of LAM. This murine model shows hyperexpression of PD-L1 in stromal cells, with PD-1 also highly expressed by activated T-cells. In this model, in vivo treatment with anti-PD1 antibody improved mouse survival.

We did not postulate that we could use Anti-PD1 to be both effective on NSCLC and LAM. The fact that there was no other potentially effective treatment for the cancer and data suggesting a theoretical effect on LAM made us decide to use it hypothesizing that there could be a decreased potential risk of flare up in comparison with other type of interstitial lung disease.

In the current case, immunotherapy proved highly successful in managing the NSCLC tumor that occurred upon LAM follow-up, with both a significantly prolonged partial response and an acceptable safety profile (Grade II hypothyroidism). However, immunotherapy did not significantly impact LAM itself, as shown by CT and functional respiratory testing. Though highly immunogenic, the LAM lung microenvironment is not similar to the melanoma tumor microenvironment. Immune cell infiltrates within LAM lungs are predominantly macrophagic in nature, whereas melanoma exhibits rather T-cell subsets or dendritic cells [12]. Such differences could explain why immunotherapy LAM treatment may fail despite immunogenic melanocyte antigens being expressed by LAM cells.

LAM treatment is based on mTOR inhibition therapies targeting the mTOR pathway, such as sirolimus [15]. We have been able so far to safely combine immunotherapy and sirolimus for at least 10 months, in spite of the tumor progression risk owing to sirolimus-induced immunosuppression. To date, the tumor response is still responding.

Since LAM is a very rare condition, its association with lung cancer has only been reported in very few cases. This association may still be purely incidental. To the best of our knowledge, however, we have described the first case of sporadic LAM with NSCLC and immunotherapy, with both encouraging tumor response and survival.

## Conclusion

Sporadic LAM is a rare condition, and its association with lung cancer has only rarely be reported to date. With a clinical and imaging pattern of interstitial lung disease, LAM deemed to display a neoplasm-like molecular pathogenesis. The use of immune checkpoint inhibitors for treating NSCLC in this patient proved safe without any serious adverse event or LAM flare up, along with a good tumor response. Combining nivolumab and sirolimus was well tolerated, without impacting efficacy nivolumab efficacy for this patient. However, if such a therapeutic choice is made in LAM patients, a close follow-up is clearly required in order to ensure the absence of LAM flare up or any toxicity.”

## Abbreviations

CK: Cytokeratin
CT: computed tomography
Gp100: Glycoprotein 100
HMB45: Human melanoma black 45
LAM: Lymphangioleiomyomatosis
MART-1: Melanoma associated antigen recognized by T-Cells
mTOR: Mammalian target of rapamycin
NSCLC: Non Small cell Lung Cancer
PD-1: Programmed death 1
PDL-1: Programmed death Ligand 1
PEComas: Perivascular Epitheliod Cell Tumors
PETTDM: Positon emitting tomography
TTF-1: Thyroid Transcription Factor 1
